# Synthesis of a Novel Polyethoxysilsesquiazane and Thermal Conversion into Ternary Silicon Oxynitride Ceramics with Enhanced Thermal Stability

**DOI:** 10.3390/ma10121391

**Published:** 2017-12-05

**Authors:** Yoshiaki Iwase, Yoji Horie, Yusuke Daiko, Sawao Honda, Yuji Iwamoto

**Affiliations:** 1 Applied Research Laboratory, General Center of Research and Development, Toagosei Co., Ltd., 8, Showa-cho, Minato-ku, Nagoya 455-0026, Japan; yoshiaki_iwase@mail.toagosei.co.jp (Y.I.); youji_horie@mail.toagosei.co.jp (Y.H.); 2 Department of Life Science and Applied Chemistry, Graduate School of Engineering, Nagoya Institute of Technology, Gokiso-cho, Showa-ku, Nagoya 466-8555, Japan; daiko.yusuke@nitech.ac.jp (Y.D.); honda@nitech.ac.jp (S.H.)

**Keywords:** silicon oxynitride, amorphous state, thermal stability, Polymer-Derived Ceramics (PDCs)

## Abstract

A novel polyethoxysilsesquiazane ([EtOSi(NH)_1.5_]_n_, EtOSZ) was synthesized by ammonolysis at −78 °C of ethoxytrichlorosilane (EtOSiCl_3_), which was isolated by distillation as a reaction product of SiCl_4_ and EtOH. Attenuated total reflection-infra red (ATR-IR), ^13^C-, and ^29^Si-nuclear magnetic resonance (NMR) spectroscopic analyses of the ammonolysis product resulted in the detection of Si–NH–Si linkage and EtO group. The simultaneous thermogravimetric and mass spectrometry analyses of the EtOSZ under helium revealed cleavage of oxygen-carbon bond of the EtO group to evolve ethylene as a main gaseous species formed in-situ, which lead to the formation at 800 °C of quaternary amorphous Si–C–N with an extremely low carbon content (1.1 wt %) when compared to the theoretical EtOSZ (25.1 wt %). Subsequent heat treatment up to 1400 °C in N_2_ lead to the formation of X-ray amorphous ternary Si–O–N. Further heating to 1600 °C in N_2_ promoted crystallization and phase partitioning to afford Si_2_N_2_O nanocrystallites identified by the XRD and TEM analyses. The thermal stability up to 1400 °C of the amorphous state achieved for the ternary Si-O-N was further studied by chemical composition analysis, as well as X-ray photoelectron spectroscopy (XPS) and ^29^Si-NMR spectroscopic analyses, and the results were discussed aiming to develop a novel polymeric precursor for ternary amorphous Si–O–N ceramics with an enhanced thermal stability.

## 1. Introduction

Silicon oxynitride (Si_2_N_2_O) is a unique crystalline compound in the silica (SiO_2_)-silicon nitride (Si_3_N_4_) binary system, and Si_2_N_2_O ceramics exhibit attractive properties for its structural application, such as low theoretical density with high hardness and low thermal expansion coefficient [1], low thermal conductivity [2], excellent oxidation resistance up to 1600 °C, and high temperature strength without degradation up to 1400 °C [3]. Moreover, the low dielectric constant and loss of the porous Si_2_N_2_O material [4] is attractive as ceramic insulators. 

Crystalline Si_2_N_2_O can be produced through the following routes: (i) high-temperature solid state reaction of Si_3_N_4_ with SiO_2_ [5]; (ii) nitridation of a mixture of Si and SiO_2_ [6]; (iii) carbothermal reduction nitridation by reacting mixtures of carbon and SiO_2_ under flowing nitrogen [7,8]; and, (iv) self-propagating high-temperature synthesis [9,10]. Similar to Si_3_N_4_, Si_2_N_2_O decomposes close to the sintering temperature, and generally hot-pressing is required for fabricating fully dense Si_2_N_2_O ceramics [2,11].

On the other hand, nonstoichiometric silicon oxynitride (SiO_*x*_N_*y*_) films can be fabricated by plasma enhanced chemical vapour deposition (PECVD) [12,13,14,15,16,17,18,19]. In addition to better dielectric properties, SiO_*x*_N_*y*_ films show excellent optical properties. The optical losses in SiO_*x*_N_*y*_ films are significantly low, and the refractive index of the films can be controlled in the wide range from 1.45 for SiO_2_ to 2.0 for Si_3_N_4_ [12], which can offer potential applications, such as antireflective coatings [13] and waveguide layers [14,15,16]. Moreover, SiO_*x*_N_*y*_ films exhibit sufficient thermal and chemical stabilities, and can thus be expected to be used for electronic devices [13,17] and buffer layers [18,19].

Recently, increasing attention has been directed to the synthesis of silicon-based non-oxide ceramics through the Polymer-Derived Ceramics (PDCs) route [20,21]. Potential advantages of this route are lower processing temperatures, easy purification of starting polymers, and, thus, the effective reduction of impurities in the final ceramic product, and formation of novel inorganic-organic hybrids, and metastable amorphous ceramics that cannot be produced by conventional powder processing methods. Moreover, polymerization and cross-linking provide a means to vary the specific properties of the pre-ceramic compounds, such as solubility, fusibility, or viscosity, extensively providing the versatility in processing and shaping capabilities, including thin film and long fiber syntheses that are similar to that successfully achieved with polymer materials. In this route, copolymers of the methylcyclosiloxanes and methylcyclolosilazanes [22], poly(Si-isocyanato-Si-methylpolysilazane) [23], and polysilyloxycarbodiimide [24] were synthesized and successfully converted to both amorphous SiO_*x*_N_*y*_ and crystalline Si_2_N_2_O. Perhydropolysilazane (–SiH_2_–NH–) was also found as a useful starting polymer to fabricate dense and hydrophilic SiO_*x*_N_*y*_ bond coat in a double-layer EBC system, which showed outstanding performance in long-term static oxidation tests at 800 °C [25].

In this study, a novel polyethoxysilsesquiazane was designed and synthesized as a single source precursor for the ternary Si–O–N ceramic system. In addition to several spectroscopic analyses, the polymer to amorphous SiO_*x*_N_*y*_ conversion process was in-situ monitored by the simultaneous thermogravimetric and mass spectrometry analyses. Then, the crystallization and phase partitioning behavior of the polymer-derived amorphous SiO_*x*_N_*y*_ was discussed from a viewpoint to develop a novel polymeric precursor for ternary amorphous Si-O-N ceramics with an enhanced thermally stability.

## 2. Experimental Section

### 2.1. Precursor Synthesis

The handling of all the reagents and products in this study was performed under inert atmosphere of pure nitrogen (N_2_). Polyethoxysilsesquiazane ([EtOSi(NH)_1.5_]_n_, EtOSZ) was synthesized via simple 2-steps reaction (Equations (1) and (2)).

(1) Synthesis of ethoxytrichlorosilane (EtOSiCl_3_)

(1) $${\mathrm{SiCl}}_{4}+{\mathrm{CH}}_{3}{\mathrm{CH}}_{2}\mathrm{OH}\;\underset{\;r.t.\;}{\to }\;{\mathrm{EtOSiCl}}_{3}$$

A 1 L four-neck round-bottom flask equipped with a dropping funnel, a magnetic stirrer, and a septum, was charged with tetrachlorosilane (500 g, 2.94 mol, Wako Pure Chemicals Industry, Osaka, Japan). Through the funnel, ethanol (99.5%, 172 mL, 2.94 mol, Wako Pure Chemicals Industry) was added dropwise at room temperature over 2 h. The mixture was then stirred at room temperature for additional 1 h. After distillation, 316 g of EtOSiCl_3_ was obtained at 102 °C/760 mmHg as a colorless liquid. The purity of the distillated EtOSiCl_3_ was monitored by gas chromatography (GC) analysis and the EtOSiCl_3_ fraction with the purity higher than 95% was collected and used for next reaction.

(2) Synthesis of EtOSZ

(2) $${\mathrm{EtOSiCl}}_{3}\;\overset{NH_{3}}{\underset{-78\;{}^{\circ}C\;\mathrm{to}\;r.t.}{\to }}\;{\left[{\mathrm{EtOSi}(\mathrm{NH})}_{1.5}\right]}_{n}$$

A 500 mL four-neck round-bottom flask equipped with a cold finger condenser, mechanical stirrer and a condenser topped with a gas outlet tube was charged with EtOSiCl_3_ (24.2 g, 0.135 mol), and freshly dried tetrahydrofuran (THF, 250 mL, Wako Pure Chemicals Industry, Osaka, Japan), and cooled to −78 °C.

Gaseous pure ammonia (NH_3_, >99.9%, Sumitomo Seika Chemicals, Osaka, Japan) at a flow rate of 500 mL/min was bubbled into the solution for 1 h through a glass tube. The suspension was stirred at −78 °C for additional 1 h, and then allowed to warm up to room temperature overnight as the excess of NH_3_ evaporated. The NH_4_Cl precipitate was then filtered off under N_2_ pressure, and was washed with fresh THF under N_2_ atmosphere. The filtrate was transferred into a 500 mL round-bottom flask and removed the solvent under vacuum at 40 °C to afford EtOSZ (12.2 g, 0.128 mol) as colorless solid. The yield was 95%.

### 2.2. Pyrolysis and Heat Treatment

The synthesized EtOSZ was placed on an alumina tray and pyrolyzed in a quartz tube furnace under flowing N_2_ (200 mL/min) by heating from room temperature up to 800 °C with a heating rate of 5 °C/min, maintaining the temperature at 800 °C for an additional 1 h and finally furnace cooling down to room temperature to give a product as a slightly brown solid.

The pyrolyzed sample was ground to fine powders using a mortar and a pestle. The powdered sample was placed on a BN plate within a BN crucible and was heat-treated in a graphite resistance-heated furnace (Model High Multi 5000, Fujidempa Kogyo, Osaka, Japan) under vacuum from room temperature to 500 °C. Then, N_2_ gas was introduced into the furnace at 500 °C and the temperature was increased to 1400, 1600, or 1800 °C, and was held for an additional 1 h. The heating rate was 10 °C /min. The N_2_ pressures that were applied in this heat treatment procedure were 196 kPa between 500 °C and 1200 °C, 392 kPa between 1200 and 1600 °C and 980 kPa between 1600 and 1800 °C. After the heat treatment, the sample was cooled down to room temperature in the furnace. 

### 2.3. Characterizations

^13^C and ^29^Si solid state nuclear magnetic resonance (NMR) spectra for the as-synthesized EtOSZ polymer and its heat-treated powdered materials were acquired using magic angle spinning (MAS), with a rotation frequency of 15 kHz (Model ECA-400, JEOL, Tokyo, Japan) at room temperature. The resonance frequencies for the ^13^C- and ^29^Si-NMR spectra that were recorded in this study were 100 and for 79.5 MHz, respectively. The chemical shifts of the peak signals in the ^13^C- and ^29^Si-NMR spectra were quoted relative to the signals of adamantine (29.5 pm) and 3-(trimethylsilyl) propionic acid sodium salt (2 ppm), respectively. 

The Attenuated Total Reflection-Infra Red (ATR-IR) spectra were recorded on the as-synthesized and pyrolyzed EtOSZ with a diamond prism under an incidence angle of 45° (Model Spectrum 100, Perkin Elmer, Waltham, MA, USA). 

The thermal behaviors up to 1000 °C were studied by thermogravimetric/differential thermal analysis (TG/DTA) in air or N_2_ (Model TG-DTA 6300, Hitachi High Technologies Ltd., Tokyo, Japan), and simultaneous TG-mass spectrometry (MS) analyses (Model STA7200, Hitachi High Technologies Ltd., Tokyo, Japan/Model JMS-Q1500 GC, JEOL, Tokyo, Japan). The measurements were performed under flowing helium (100 mL/min) with a heating rate of 10 °C/min.

Elemental analyses were performed on the pyrolyzed or heat-treated samples for oxygen, nitrogen, and hydrogen (inert-gas fusion method, Model EMGA-930, HORIBA, Ltd., Kyoto, Japan), and carbon (non-dispersive infrared method, Model CS844, LECO Co., St. Joseph, MI, USA). The silicon content in the samples was calculated as the difference of the sum of the measured C, N, O, and H content to 100 wt %.

X-ray diffraction (XRD) measurements were performed on pyrolyzed or heat-treated samples (Model X’pert Pro α1, Philips Ltd., Amsterdam, The Netherlands). 

X-ray photoelectron spectroscopy (XPS) measurements were performed on heat-treated samples (Model PHI-5000, Ulvac-phi, Kanagawa, Japan).

Crystallization behavior of the EtOSZ-derived amorphous silicon oxynitride (Si–O–N) materials was observed by using a transmission electron microscope (TEM, Model 2010, JEOL, Tokyo, Japan, operating at 200 kV, camera length = 80 cm).

## 3. Results and Discussion

### 3.1. Chemical Structure of EtOSZ

**Figure 1:**
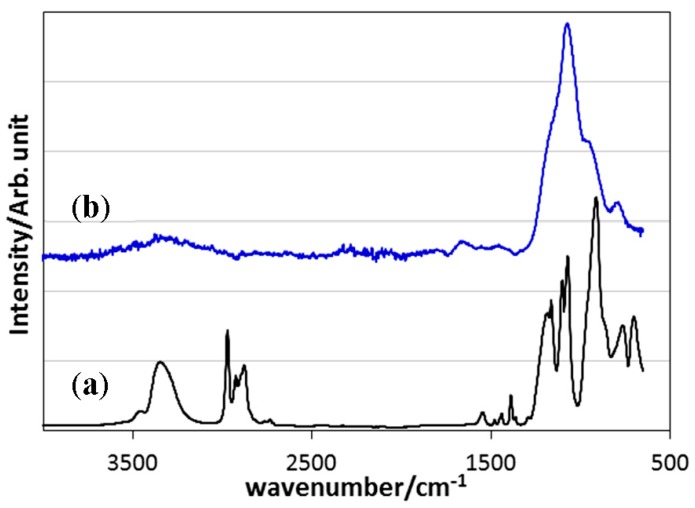
Attenuated Total Reflection-Infra Red (ATR-IR) spectra of (**a**) as-synthesized and (**b**) 800 °C-pyrolyzed polyethoxysilsesquiazane (EtOSZ).

The chemical structure of the synthesized EtOSZ was initially studied by the ATR-IR spectroscopic analysis. As shown in Figure 1, the spectrum of the as-synthesized sample exhibited characteristic absorption bands at 3350 (broad), 2800–3000, and 1070 cm^−1^, attributed to νN–H, νC–H, and δN–H that were involved in Si–NH–Si unit [26], respectively. 

**Figure 2:**
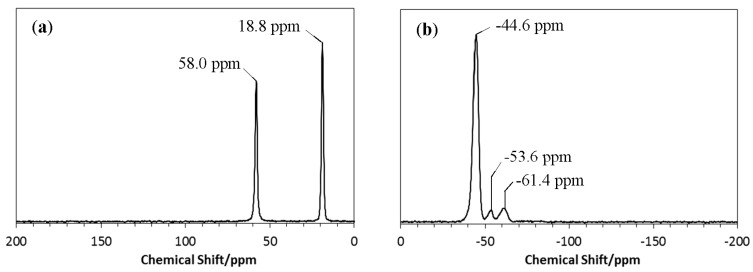
(**a**) ^13^C- and (**b**) ^29^Si-NMR spectra of EtOSZ.

To identify the chemical structure of the EtOSZ in more details, ^13^C- and ^29^Si-NMR spectroscopic analyses were performed in solid state. Results were shown in Figure 2. The ^13^C-NMR spectrum presented two sharp signals at 58.0 and 18.8 ppm, assigned to methylene (CH_2_) unit, and terminate methyl (CH_3_) unit in the ethoxy (OCH_2_CH_3_) group, respectively [27]. On the other hand, the corresponding ^29^Si-NMR spectrum exhibited a strong single signal at −44.6 ppm that was assigned to SiO(NH)_3_ unit of the reaction product, EtOSZ ([CH_3_CH_2_OSi(NH)_1.5_]_n_). The weak signals at −53.6 and −61.4 ppm were thought to be attributed to by-products that could not be removed by the distillation after the alkoxylation of SiCl_4_ (Equation (1)). The signals at −53.6 and −61.4 ppm were assigned to (EtO)_2_–Si–(NH)_2_ (linear or cyclic) and (EtO)_3_–Si–NH, respectively. As mentioned in the experimental section, the purity of the distilled EtOSiCl_3_ was higher than 95%, and the total amount of these by-products was small.

These results supported that the two-steps reaction route that was investigated in this study is useful for the synthesis of EtOSZ.

### 3.2. Conversion to Inorganic Compound

**Figure 3:**
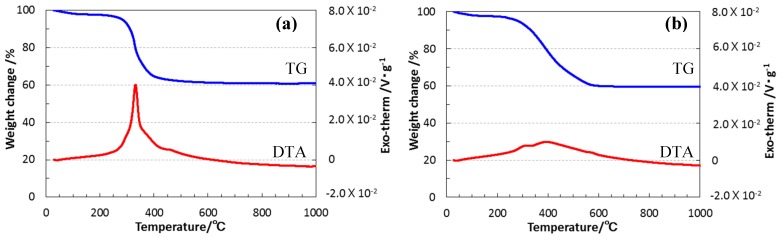
Thermogravimetric/differential thermal analysis (TG-DTA) curves for EtOSZ measured (**a**) in air and (**b**) in N_2_.

To study the thermal property of the EtOSZ that was synthesized in this study, the TG/DTA analyses both in air and in N_2_ atmosphere were, respectively, performed. The results are shown in Figure 3.

In air (Figure 3a), the sample showed a slight weight loss of approximately 2.5% up to 200 °C, which could be due to the residual solvent. Then, a main weight loss was observed at around 300 to 600 °C, with a distinct exothermic peak that was centered at 321 °C, typical for the combustion of organic molecules. The final recovery rate (ceramic yield) at 1000 °C was 60%. This yield was assumed to be recognized as a result of the combustion of organic substituents and subsequent oxidation of Si atom in the EtOSZ to yield silica (Equation (3)), since the observed mass loss was consistent with the weight difference between the molecular unit of EtOSZ (CH_3_CH_2_OSi(NH)_1.5_) and the molecular weight of silica (SiO_2_).

CH_3_CH_2_OSi(NH)_1.5_ + 7.75O_2_ → SiO_2_ + 2CO_2_↑ + 1.5NO_2_↑ + 3.25H_2_O↑ (3)

Under flowing N_2_ (Figure 3b), EtOSZ also showed a main weight loss at the same temperatures ranging from 300 to 600 °C, and the ceramic yield at 1000 °C was 58%, close to that achieved in air, while a weak and very broad exothermic peak appeared at 50 to 700 °C. 

In the ATR-IR spectrum for the sample after pyrolysis at 800 °C in N_2_ (Figure 1), the characteristic peaks due to the organic substituents disappeared and the spectrum was similar to that of silica composed of a weak and broad band at 3000–3700 cm^−1^ and a strong band centered around 1010 cm^−1^ assigned to intermolecular hydrogen-bonded Si–OH groups and ν*_as_* Si–O in Si–O–Si linkage [27,28].

**Figure 4:**
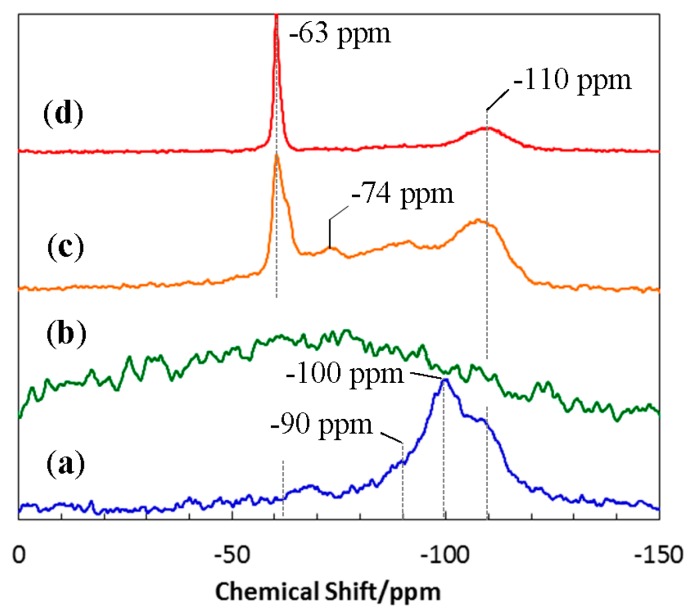
^29^Si-NMR spectra of (**a**) 800 °C-pyrolysed EtOSZ and the samples after the subsequent heat treatment at (**b**) 1400 °C; (**c**) 1600 °C; and (**d**) 1800 °C in N_2_.

To study the chemical structure of the 800 °C-pyrolyzed EtOSZ in more detail, ^29^Si-NMR spectroscopic analysis was performed. The result was shown in Figure 4.

One broad peak at around −100 ppm was deconvoluted to three broad peaks centered at −110, −100, and −90 ppm that were assigned to silicon tetrahedral units of SiO_4_ [29], HO–SiO_3_ (Q3) [30], and SiO_3_N [31,32], respectively. 

Chemical composition of the 800 °C-pyrolyzed EtOSZ was listed in Table 1. As a reference data, the theoretical composition of the as-synthesized EtOSZ was also listed in this table. 

**Figure 5:**
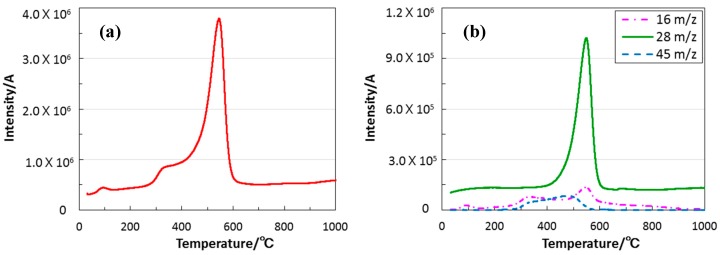
The relationships of heating temperature and evolution of gaseous species; (**a**) total ion current chromatogram (TICC); and (**b**) Constituents of evolved gaseous species.

In spite of the pyrolysis under inert atmosphere of N_2_, the carbon content remarkably decreased from 25.1 to 1.1 wt %, and the resulting C/Si atomic ratio was 0.05. The N/Si atomic ratio also decreased from 1.5 to 0.5, while the O/Si atomic ratio was 1.1, and close to that of the ideal EtOSZ (1.0). Then, TG-MS analysis was performed on the as-synthesized EtOSZ under He atmosphere. The results were summarized and are shown in Figure 5. 

The TG-curve that was measured in He was quite similar to the previous one in N_2_ (Figure 3b), and the gaseous species formed in-situ were mainly detected during the main weight loss at 300 to 600 °C. As-shown in Figure 5a, the total ion current chromatogram (TICC) spectrum showed a broad bimodal signal composed of a weak peak at 300 to 400 °C, and a dominant one at 400 to 600 °C. The simultaneous MS analysis resulted in the detection of three kinds of gaseous species at the *m*/*z* ratios of 45, 28, and 16 (Figure 5b). 

The gaseous species, *m*/*z* ratios at 45 and 16 were assigned to SiNH_3_^+^ and NH_2_^+^, respectively. These fragment ions could be due to the partial decomposition of the silsesquiazane linkage, which leading to the lower nitrogen content observed for the 800 °C-pyrolysed sample. On the other hand, the *m*/*z* ratio at 28 could be assigned to ethylene (CH_2_ = CH_2_^+^), and it turned out that the dominant thermal decomposition reaction of the EtOSZ was the C-O bond cleavage of the ethoxy group to afford CH_2_ = CH_2_^+^ as the main hydrocarbon gaseous product (Equation (4)), which was leading to the remarkable decrease in carbon content:

–Si–O–CH_2_–CH_3_ → –SiO^●+^ + CH_2_ = CH_2_^+^ + 1/2H_2_ (4)

### 3.3. Crystallization Behavior of EtOSZ-Derived Amorphous Si-O-N in N_2_

**Figure 6:**
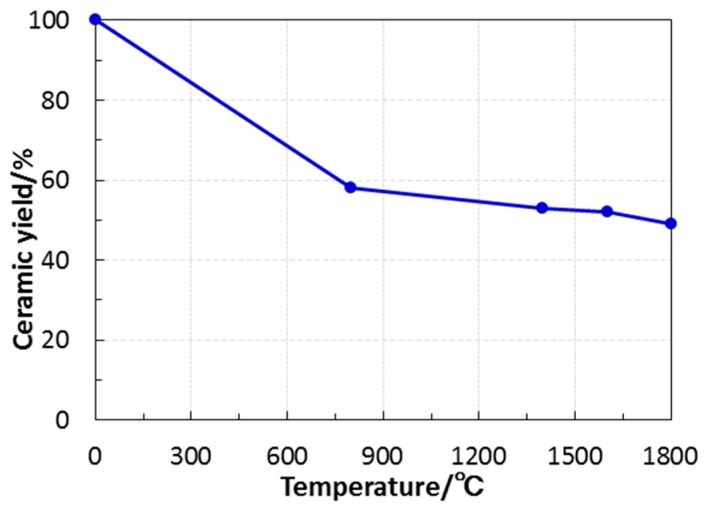
Weight change of EtOSZ during pyrolyisis up to 800 °C followed by heat treatment up to 1800 °C in N_2_.

Polymer-derived ternary and quaternary amorphous Si–(M)–C–N (M = B, Ti, etc.) show a unique high-temperature stability in terms of restricting crystallization and subsequent phase partitioning under an inert atmosphere of N_2_ or Ar [20,21]. On the other hand, very limited study has been done for the crystallization behavior of polymer-derived ternary Si–O–N or quaternary Si–O–C–N systems [22,24]. Therefore, high-temperature crystallization behavior above 1000 °C of the present EtOSZ-derived amorphous Si–O–C–N was further studied. To restrict Si–N bond cleavage, as mentioned in the experimental section, the additional heat treatment at 1200 to 1400 °C, and above 1400 °C, were performed under the N_2_ atmospheres of 392 and 980 kPa, respectively. As shown in Figure 6, the total weight loss up to 1800 °C of the 800 °C-pyrolyzed sample was approximately 9%, and was found to be much lower than that during pyrolysis up to 800 °C (42 %, Figure 3b). 

**Figure 7:**
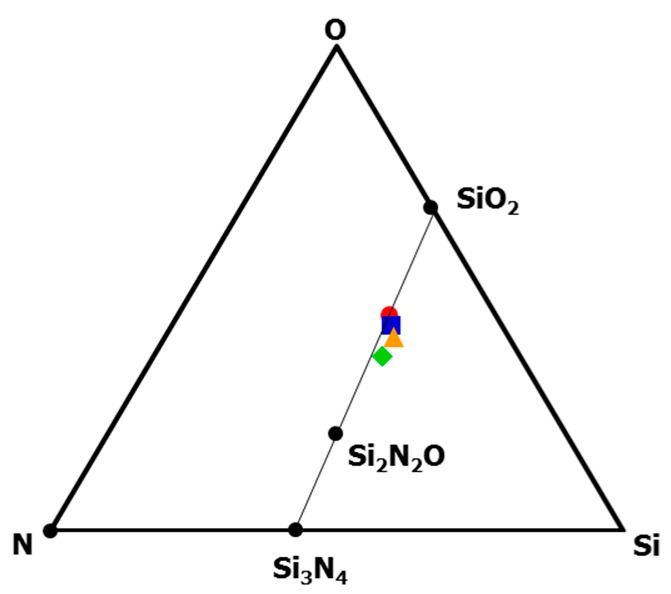
Compositions of EtOSZ-derived samples after pyrolysis at 800 °C (■), and subsequent heat treatment at 1400 °C (0.3Si_2_N_2_O: 0.3SiO_2_: 0.1Si, ♦), 1600 °C (0.25Si_2_N_2_O: 0.375SiO_2_: 0.125Si, ▲), and 1800 °C (0.2Si_2_N_2_O: 0.4SiO_2_: 0.2Si, ●) in N_2_.

To study the composition change during the heat treatment up to 1800 °C, elemental analyses were performed on the heat-treated samples. The results were summarized in Table 1. Since the carbon and hydrogen contents in the heat-treated samples were negligibly small (below 0.5%), the compositions of the samples were also plotted in the ternary Si–O–N phase diagram (Figure 7). As a reference sample, 800 °C-pyrolyzed sample was also plotted in this diagram without counting the contents of carbon and hydrogen. Upon heating to 1400 °C, carbon in the EtOSZ-derived Si–O–C–N could be almost spent out to yield gaseous CO_*x*_ (x = 1, 2). The resulting composition of the 1400 °C-heated sample that was located close to the tie line between Si_2_N_2_O and SiO_2_. Then, above 1400 °C, the position shifted toward SiO_2_ along the Si_2_N_2_O-SiO_2_ tie line due to the decreasing nitrogen content. 

**Figure 8:**
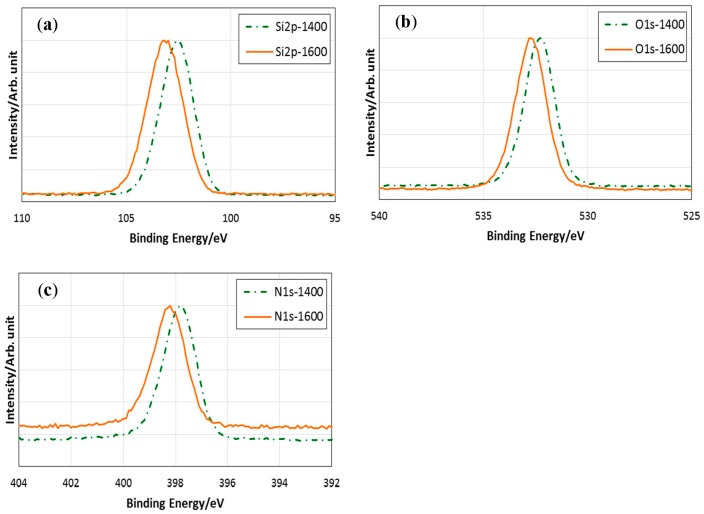
X-ray photoelectron spectroscopy (XPS) spectra for 1400 °C- and 1600 °C-heat treated samples; (**a**) Si2*p*; (**b**) O1*s*; and (**c**) N1*s* regions.

Figure 8 shows the results of XPS analysis for the heat-treated samples. The 1400 °C heat-treated sample had a Si2*p* binding energy of 102.6 eV, intermediate to SiO_2_ (103.4 eV) and Si_3_N_4_ (101.7 eV) [33]. This value was closed to that reported for amorphous silicon oxynitride (102.4 eV) [34] and was consistent with the random bonding model for the partial replacement of oxygen in the SiO_4_ tetrahedron by nitrogen, causing the lower binding energies of the Si2*p* binding energy [35]. 

After the heat treatment at 1600 °C, the Si2*p* binding energy was centered at 103 eV (Figure 8a). By the 1600 °C-heat treatment, the peak center of the N1*s* binding energy also shifted from 397.8 to 398.2 eV (Figure 8c). These peaks that shift behaviors toward higher binding energy were consistent with the decrease in nitrogen content (Table 1, Figure 7), as reported for the ternary amorphous silicon oxynitride (Si–O–N) by Weeren et al. [32]. The peak centers of the O1*s* binding energies for the 1400 °C- and 1600 °C-heated samples were 532.2 and 532.7 eV, respectively (Figure 8b). These values were also compatible with those reported for SiO_2_ and amorphous silicon oxynitride having nitrogen content ranging from 0 to 47 at %. 

These results indicate that the EtOSZ that was investigated in this study could be converted to a unique oxygen rich amorphous silicon oxynitride (Si–O–N) by pyrolysis at 800 °C, followed by heat treatment at 1400 °C in N_2_. 

**Figure 9:**
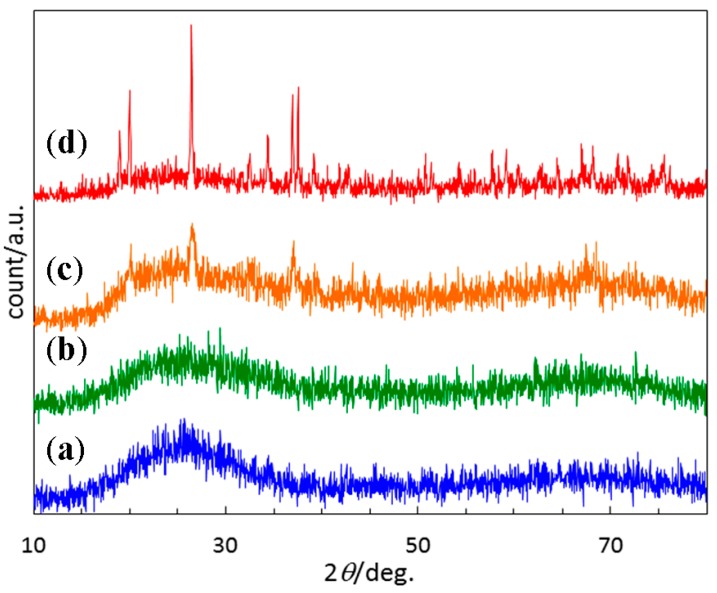
X-ray diffraction (XRD) patterns of (**a**) 800 °C-pyrolysed EtOSZ and the samples after the subsequent heat treatment at (**b**) 1400 °C; (**c**) 1600 °C; and (**d**) 1800 °C in N_2_.

**Figure 10:**
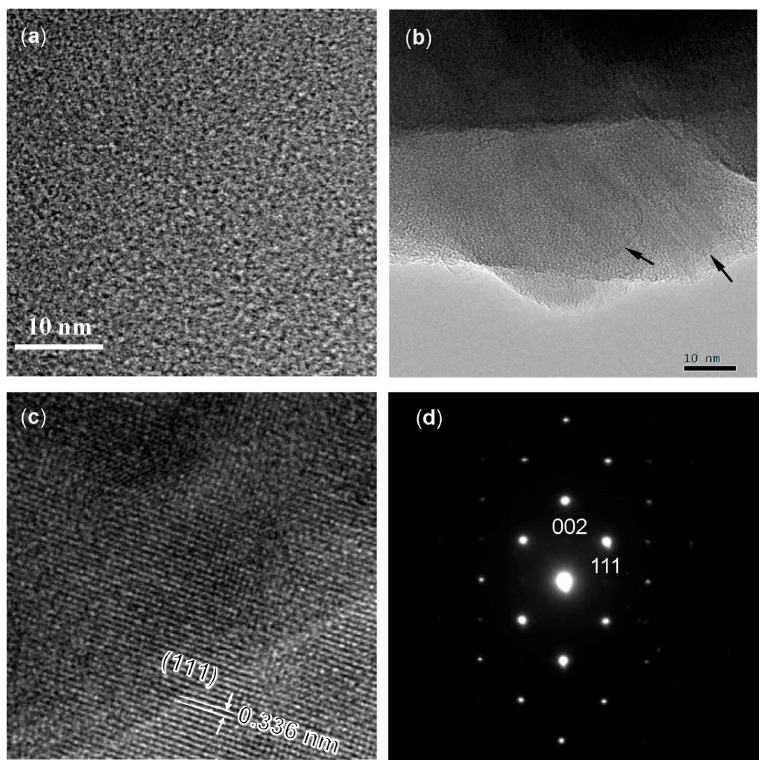
Typical TEM images showing (**a**) X-ray amorphous Si-O-N after heat treatment at 1400 °C; (**b**) nanocrystallites (indicated by arrows) formed in-situ within the amorphous matrix by heating to 1600 °C; (**c**) high resolution TEM (HRTEM) image; and, (**d**) selected area electron diffraction (SAEDP) obtained for the nanocrystallite shown in (**b**).

As shown in Figure 9, this material was found to keep X-ray amorphous up to 1400 °C. Then, after the 1600 °C-heat treatment, the sample began to show a diffraction pattern that was identical to crystalline Si_2_N_2_O (JCPDS 47-1627) [36], and the intensity of the Si_2_N_2_O diffraction peaks increased to some extent by the 1800 °C-heat treatment. Actually, as shown in Figure 10a, the 1400 °C-heated sample exhibited a futureless structure, which is typical for amorphous compounds. Then, after the 1600 °C-heat treatment, some crystallites of several ten nanometers in size were observed within the amorphous matrix (marked by arrows in Figure 10b). The inter planer spacing that was observed for the nanocrystallite formed in-situ was measured to be 0.336 nm, which was corresponding to (111) plane of orthorhombic Si_2_N_2_O [36] (Figure 10c), and the selected area electron diffraction (SAED) pattern that was obtained from the nanocrystallite could be also indexed as (110) orthorhombic Si_2_N_2_O [36] (Figure 10d). 

To study the unique thermal stability up to 1400 °C of the EtOSZ-derived oxygen-rich amorphous Si–O–N, ^29^Si-NMR spectroscopic analysis was performed on the heat-treated samples. The resulting spectra were shown in Figure 4. The 1400 °C-heated sample showed a broad line without peaks that were characteristic for the short-range ordering of tetrahedral Si coordination, such as SiO_4_, HO–SiO_3_, and SiO_3_N. Then, after the 1600 °C-heating, the sample exhibited a relatively strong peak at −63 ppm that was attributed to SiON_3_ unit [31,32], which composing crystalline Si_2_N_2_O along with three broad peaks at around −110, −90 and −74 ppm assigned to SiO_4_, SiO_3_N, and SiO_2_N_2_ [31,32], respectively. Finally, after the 1800 °C-heating, the spectrum tuned to be composed of a distinct peak of SiON_3_ unit at −63 ppm and a broad peak centered at -110 ppm assigned to SiO_4_ unit, which was indicating that the Si_2_N_2_O and SiO_2_ two-phase partitioning was almost completed. The 800 °C-pyrolysis of EtOSZ resulted in the formation of an inorganic amorphous network that was mainly composed of SiO_4_ and HO–SiO_3_ units. Moreover, the subsequent heat treatment in N_2_ lead to the structural rearrangement to afford random network prior to the formations of SiON_3_ and SiO_2_ units that are essential for the nucleation and crystallization of thermodynamically stable Si_2_N_2_O and SiO_2_, respectively. As a result, the EtOSZ-derived oxygen rich Si–O–N could keep amorphous state up to 1400 °C in N_2_.

## 4. Summary

In this study, a novel preceramic polymer, polyethoxysilsesquiazane (EtOSZ), was designed and synthesized for ternary Si–O–N ceramic system. Chemical structure and the thermal behavior up to 1600 °C in N_2_ of the synthesized EtOSZ was investigated. The results can be summarized as follows:

(1) ATR-IR, ^13^C- and ^29^Si-NMR spectroscopic analyses revealed that the synthesized polymer was composed of EtOSi(NH)_3_ unit, and polyethoxysilsesquiazane was successfully synthesized in a good yield via simple two-steps reaction, stoichiometric reaction of SiCl_4_ with EtOH to afford EtOSiCl_3_, followed by ammonolysis at −78 °C.
(2) Under an inert atmosphere, thermal decomposition of EtOSZ mainly proceeded at around 200 to 600 °C, and the resulting ceramic yield after heating to 1000 °C was 58%.
(3) The simultaneous TG-MS analyses for the thermal decomposition identified ethylene as a main gaseous species that was formed in-situ, and it was clarified that cleavage of oxygen-carbon bond of the EtO group in the EtOSZ contributed to the formation of the quaternary amorphous Si–O–C–N with extremely low carbon content (1.1 wt %) after pyrolysis at 800 °C in N_2_.
(4) Additional heat treatment up to 1400 °C of the 800 °C-pyrolyzed EtOSZ resulted in the further reduction of the carbon content to afford oxygen rich Si–O–N amorphous ceramics.
(5) The EtOSZ-derived Si–O–N was found to keep an amorphous state up to 1400 °C in N_2_, then Si_2_N_2_O crystallization started during heat treatment from 1400 to 1600 °C.

The enhanced thermal stability that was achieved for the EtOSZ-derived amorphous Si–O–N in this study could be due to the following structural changes in a short range order: Formation of amorphous network mainly composed of SiO_4_ and HO–SiO_3_ units at 800 °C, followed by structural rearrangement to afford random amorphous network at around 1400 °C prior to the formation of SiON_3_ and SiO_4_ units that are essential for the formation of Si_2_N_2_O nanocylstallites-dispersed amorphous SiO_2_ composite. 

## Figures and Tables

**Table 1:** Composition change of EtOSZ through 800 °C-pyrolysis and subsequent heat treatment up to 1800 °C in N_2_.

Sample	Composition/wt %					Empirical Ratio
	Si	C	O	N	H	
As-synthesized	29.3	25.1	16.8	22.0	6.80	Si_1.0_C_2.0_O_1.0_N_1.5_H_6.5_
800 °C-pyrolysed	51.3	1.10	32.4	13.5	1.69	Si_1.0_C_0.05_O_1.1_N_0.5_H_0.9_
1400 °C-heat treated	54.7	0.41	29.4	15.5	0.03	Si_1.0_C_0.01_O_0.9_N_0.6_H_0.0_
1600 °C-heat treated	55.1	0.30	31.8	12.8	0.01	Si_1.0_C_0.01_O_1.0_N_0.5_H_0.0_
1800 °C-heat treated	56.2	0.04	33.2	10.6	0.01	Si_1.0_C_0.0_O_1.0_N_0.4_H_0.0_

## References

[1] Larker R. (1992). Reaction sintering and properties of silicon oxynitride densified by hot isostatic pressing. J. Am. Ceram. Soc..

[2] Ohashi M., Kanzaki S., Tabata H. (1988). High-Temperature Flexural Strength of Hot-Pressed Silicon Oxynitride Ceramics. J. Mater. Sci. Lett..

[3] Ohashi M., Kanzaki S., Tabata H. (1991). Processing, Mechanical Properties, and Oxidation Behavior of Silicon Oxynitride Ceramics. J. Am. Ceram. Soc..

[4] Li S.Q., Pei Y.C., Yu C.Q., Li J.L. (2009). Mechanical and Dielectric Properties of Porous Si_2_N_2_O-Si_3_N_4_ in Situ Composites. Ceram. Int..

[5] Washbum M.E. (1967). Silicon Oxynitride Refractories. Am. Ceram. Soc. Bull..

[6] Li X.M., Zhang L., Yin X.W. (2013). Study on in-Situ Reaction Synthesis and Mechanical Properties of Si_2_N_2_O Ceramic. Ceram. Int..

[7] Bolech M., Metselaar R., van Dijen F.K., Blomer F., de With G., Ramaekers P.R.J. (1987). Carbothermal Preparation of Si_2_N_2_O Powder. High Technology Ceramics.

[8] Zhou Y., Liu Q., Zhou H., Zhuang J. (2013). Yttrium Oxide-Assisted CRN Synthesis of Silicon Oxynitride Powders with Controlled Morphology. J. Am. Ceram. Soc..

[9] Pradeilles N., Record M.C., Marin-Ayral R.M., Linde A., Studenikin L.A., Grachev V.V. (2008). Influence of Thermal Conditions on the Combustion Synthesis of Si_2_N_2_O Phase. Mater. Res. Bull..

[10] Radwan M., Kashiwagi T., Miyamoto Y. (2003). New Synthesis Route for Si_2_N_2_O Ceramics Based on Desert Sand. J. Eur. Ceram. Soc..

[11] Tong Q., Wang J., Li Z., Zhou Y. (2007). Low-temperature synthesis/densification and properties of Si_2_N_2_O prepared with Li_2_O additive. J. Eur. Ceram. Soc..

[12] Kijaszek W., Oleszkiewicz W., Zakrzewski A., Patela S., Tłaczała M. (2016). Investigation of optical properties of silicon oxynitride films deposited by RF PECVD method. Mater. Sci.-Pol..

[13] Lipiński M., Kaminski A., Lelievre J.-F., Lemiti M., Fourmond E., Zięba P. (2007). Investigation of graded index SiO_*x*_N_*y*_ antireflection coating for silicon solar cell manufacturing. Phys. Status Solidi C.

[14] Mogensen K.B., Friis P., Hubner J., Petersen N., Jørgensen A.M., Telleman P., Kutter J.P. (2001). Ultraviolet transparent silicon oxynitride waveguides for biochemical microsystems. Opt. Lett..

[15] Sabac A., Gorecki C., Jozwik M., Nieradko L., Meunier C., Gut K. (2007). Technology and performances of silicon oxynitride waveguides for optomechanical sensors fabricated by plasma-enhanced chemical vapour deposition. J. Eur. Opt. Soc..

[16] Aparicio F.J., Froner E., Rigo E., Gandolfi D., Scarpa M., Han B., Ghulinyan M., Pucker G., Pavesi L. (2014). Silicon oxynitride waveguides as evanescent-field-based fluorescent biosensors. J. Phys. D Appl. Phys..

[17] Hiranaka K., Yamaguchi T. (1990). Amorphous Silicon Thin-Film Transistors with SiO_*x*_N_*y*_/SiN_*x*_ Gate Insulators. Jpn. J. Appl. Phys..

[18] Jung S., Kim J., Son H., Hwang S., Jang K., Lee J., Lee K., Park H., Kim K., Yi J. (2007). Fabrication and characterization of metal-oxide-nitride-oxynitride-polysilicon nonvolatile semiconductor memory device with silicon oxynitride (SiO_*x*_N_*y*_) as tunneling layer on glass. J. Appl. Phys..

[19] Heo S., Lee J., Kim S.H., Yun D.-J., Park J.-B., Kim K., Kim N.J., Kim Y., Lee D., Kim K.-S. (2017). Device performance enhancement via a Si-rich silicon oxynitride buffer layer for the organic photodetecting device. Sci. Rep..

[20] Kroke E., Li Y.-L., Konetschny C., Lecomte E., Fasel C., Riedel R. (2000). Silazane derived ceramics and related materials. Mater. Sci. Eng..

[21] Colombo P., Mera G., Riedel R., Soraru G.D. (2010). Polymer-derivedceramics: 40 years of research and innovation in advanced ceramics. J. Am. Ceram. Soc..

[22] Yu G.-E., Edirisinghe M., Finich D., Ralph B., Parrick J. (1995). Synthesis of silicon oxynitride from a polymeric precursor, Part IV Pyrolysis of the copolymers. J. Mater. Sci..

[23] Gunji T., Suzuki Y., Abe Y. (2000). Solid State NMR Analysis on the Conversion Process of Poly(Si-isocyanato-Si-methylpolysilazane) into Silicon Nitride Oxide. Nippon Kagaku Kaishi.

[24] Cheng H., Li Y., Kroke E., Herkenhoff S. (2013). In situ synthesis of Si_2_N_2_O/Si_3_N_4_ composite ceramics using polysilyloxycarbodiimide precursors. J. Eur. Ceram. Soc..

[25] Wang K., Günthner M., Motz G., Flinn B.D., Bordia R.K. (2013). Control of Surface Energy of Silicon Oxynitride Films. Langmuir.

[26] Seyferth D., Wiseman G., Prud’homme C. (1983). A liquid silazane precursor to silicon nitride. J. Am. Ceram. Soc..

[27] Blanco I., Bottino F.A., Cicala G., Latteri A., Recca A. (2013). A kinetic study of the thermal and thermal oxidative degradations of new bridged POSS/PS nanocomposites. Polym. Degrad. Stab..

[28] Sokri M.N.M., Onishi T., Mouline Z., Daiko Y., Honda S., Iwamoto Y. (2015). Polymer-derived amorphous silica-based inorganic-organic hybrids having alkoxy groups: Intermediates for synthesizing microporous amorphous silica materials. J. Ceram. Soc. Jpn..

[29] Levy G.C., Cargioli J.D., Axenrod T., Webb G.A. (1974). Nuclear Magnetic Resonance Spectroscopy of Nuclei Other than Protons.

[30] Engelhardt G., Jancke H., Magi M., Pehk T.J., Lippma E. (1971). Über die ^1^H-, ^13^C- und ^29^Si-NMR chemischen Verschiebungen einiger linearer, verzweigter und cyclischer Methylsiloxan-Verbindungen. J. Organomet. Chem..

[31] Dupree R., Lewis M.H., Smith M.E. (1988). High-Resolution Silicon-29 Nuclear Magnetic Resonance in the Y-Si-O-N System. J. Am. Chem. Soc..

[32] Dupree R., Lewis M.H., Smith M.E. (1989). High-Resolution NMR Study of the La-Si-Al-O-N System. J. Am. Chem. Soc..

[33] Weeren R.V., Leone E.A., Curran S., Klein L.C., Danforth S.C. (1994). Synthesis and Characterization of Amorphous Si_2_N_2_O. J. Am. Ceram. Soc..

[34] Donely M.S., Baer D.R., Stoebe T.G. (1988). Nitrogen 1s Charge Referencing for Si, N, and Related Compounds. Surf. Interface Anal..

[35] Belyi V.I., Vasilyeva L.L., Ginorker A.S., Gritsenko V.A., Repinsky S.M., Sinitsa S.P., Sinirnova T.P., Edelman F.L., Edelman F.L. (1988). Silicon Nitride in Electronics. Materials Science Monographs.

[36] Sjöberg J., Helgesson G., Idrestedt I. (1991). Refinement of the structure of Si_2_N_2_O. Acta Crystallogr..

